# Sex and onset-age-related features of excessive daytime sleepiness and night-time sleep in patients with Parkinson’s disease

**DOI:** 10.1186/s12883-021-02192-x

**Published:** 2021-04-19

**Authors:** Ming Liu, Ya-Jun Luo, Han-Ying Gu, Yi-Ming Wang, Man-Hua Liu, Kai Li, Jiao Li, Sheng Zhuang, Yun Shen, Hong Jin, Jing Chen, Cheng-Jie Mao, Chun-Feng Liu

**Affiliations:** 1 Department of Neurology and Suzhou Clinical Research Center of Neurological Diseases, the Second Affiliated Hospital of Soochow University, Suzhou, 215004 Jiangsu Province China; 2 Department of Neurology, Suqian First Hospital, Suqian, China

**Keywords:** Parkinson’s disease, Sex difference, Age at onset, Excessive daytime sleepiness, Nocturnal sleep quality

## Abstract

**Background:**

The clinical characteristics of Parkinson’s disease (PD) differ between men and women, and late- and early-onset patients, including motor symptoms and some nonmotor symptoms, such as cognition, anxiety, and depression.

**Objective:**

To explore the features of excessive daytime sleepiness (EDS) and night-time sleep quality in PD patients of different sexes and age at onset (AAO).

**Methods:**

Demographic data and clinical characteristics of 586 PD patients were collected. Epworth Sleepiness Scale (ESS) and Pittsburgh Sleep Quality Index (PSQI) were used to investigate the daytime drowsiness and nocturnal sleep. Multivariate logistic regression analysis was used to explore the risk factors of EDS and poor night-time sleep quality.

**Results:**

Sleep disorders were common in PD patients. EDS was more prominent in men than in women. There was no significant difference in ESS scores between late-onset PD (LOPD) and early-onset PD. LOPD patients had a higher probability of poor night-time sleep quality. Male sex, disease duration, and depression were risk factors for EDS. In all patients of both sexes and all AAO, depression was a risk factor for poor night-time sleep.

**Conclusion:**

More attention should be paid to sleep disorders of PD patients, especially male LOPD patients. Depression is a common risk factor for EDS and poor sleep quality in PD patients.

## Introduction

Parkinson’s disease (PD) is the second most common neurodegenerative disease, characterized by major motor symptoms such as tremor, rigidity, bradykinesia, and postural instability. The motor manifestations of PD are related to the cortico-striatal pathway dysfunction caused by degeneration of dopaminergic neurons in the nigrostriatal pathway [1]. Various nonmotor symptoms (NMSs) reflect the dysfunction outside the nigrostriatal pathway, including neuropsychiatric, cognitive, gastrointestinal and sensory symptoms, as well as sleep disorders. There is increasing evidence that NMSs may appear before motor symptoms, which may negatively affect quality of life more than motor symptoms do [2].

Sleep disorders can affect up to 90% of PD patients [3]. The pathophysiology of sleep-wake disturbances in PD is largely unknown and may be caused by multiple factors, including the effects of motor symptoms and NMSs on sleep, adverse reactions to dopaminergic medication, and degeneration of central sleep regulatory areas [4]. The sleep-wake disorders in PD include insomnia, rapid eye movement (REM) sleep behavior disorder (RBD), excessive daytime sleepiness (EDS), restless leg syndrome (RLS), periodic limb movement (PLM), and sleep apnea syndrome (SAS). Among these, EDS and poor night-time sleep are the most common types of sleep disorders in PD patients. Sleep disorders can lead to emotional and cognitive impairment, decreased quality of life, and increased risk of accidents, leading to increased morbidity and mortality in this population [5, 6].

Although most PD patients are elderly and known as late-onset PD (LOPD) patients, 5–10% of patients develop symptoms before the age of 40 or 50 years. They are called early-onset PD (EOPD) patients. Studies have revealed that sex and age at onset (AAO) are important factors affecting the clinical phenotypes of PD, not only for motor symptoms but also for NMSs. The prevalence and incidence of PD in men are higher than in women [7]. Female patients may have a more benign phenotype in the early stage [8]. However, as the disease progresses, women seem to be at higher risk of treatment-related complications, such as motor and nonmotor fluctuations, dyskinesia, and high levels of disability [9, 10]. In terms of NMSs, male patients may experience more cognitive impairment and urinary symptoms, but less emotional, apathy and pain symptoms [11, 12]. LOPD also shows more NMSs, such as cognitive dysfunction, autonomic dysfunction, and gastrointestinal symptoms [13, 14].

There are few studies on sleep disorders in patients with PD of different sexes and AAO, and the results are controversial. It has been suggested that sleep disorders are more common in female PD patients, but a recent multicenter study found that the incidence of sleep disorders was similar in men and women [15]. Female patients are more likely to have symptoms such as difficulty falling asleep and RLS [16]. Male patients are more likely to have EDS and more aggressive behavior with RBD [4, 17, 18].

The purpose of this study was to investigate the clinical characteristics of EDS and night-time sleep quality of PD patients of different sexes and AAO by Epworth Sleepiness Scale (ESS) and Pittsburgh Sleep Quality Index (PSQI), and to explore the possible influencing factors.

## Materials and methods

### Patients

A total of 586 PD patients were enrolled in this study at the Second Affiliated Hospital of Soochow University from January 2014 to December 2019. Subjects were diagnosed with idiopathic PD according to the UK PD Society Brain Bank Clinical Diagnostic Criteria [19]. Patients who were able to complete a detailed evaluation were enrolled in this study. Subjects were excluded if they had a secondary parkinsonism syndrome, atypical parkinsonian syndrome, traumatic head or spine injury, malignant tumor, epilepsy, encephalitis, and global cognitive impairment (Mini-mental State Examination [MMSE] [20] score < 24). Patients are divided into EOPD (AAO ≤50 years) and LOPD (AAO > 50 years). We collected demographic and clinical data in detail, including sex, age, AAO, disease duration, daily levodopa equivalent dose (LED), motor subtype, motor fluctuation, and dyskinesia. Motor function was assessed by the Unified Parkinson’s Disease Rating Scale (UPDRS) Part III [21], and disease severity was evaluated according to the Hoehn and Yahr (H&Y) Stage [22]. To categorize motor subtypes, we evaluated the UPDRS Part III, and adopted the classification scheme described by Schiess, MC. et al. [23], thereby obtaining the tremor/akinetic-rigid (T/AR) ratio. We used portions of the UPDRS III, and obtained a tremor score (sum of items 20–21 and history of arm tremor [two items] divided by 9 [total number of subitems]) and a non-tremor score (sum of items 18, 19, 22, 27–31 divided by 12 [total number of subitems]). Based on this method, AR subjects had a ratio <  0.8, whereas tremor dominant (TD) subjects had a ratio > 1.0, and mixed subjects had a ratio range from 0.8 to 1. Hamilton Rating Scale for Depression (HRSD) was used to evaluate depression [24]. Screening of RBD was made according to RBD Screening Questionnaire (RBDSQ) [25].

### Sleep assessments

Daytime sleepiness was evaluated by the Epworth Sleepiness Scale (ESS) [26], a generic scale recommended for measuring the risk of falling asleep during daily activities in the PD population [27]. As reported, ESS shows adequate coincidence with results of multiple sleep latency test (MSLT) and overnight polysomnography (PSG), as well as SCOPA-Sleep Daytime Sleepiness (SCOPA-Sleep-DS, a PD-specific scale) scores in PD patients [28]. It contains eight questions, each with a score ranging from 0 (would never doze) to 3 (high chance of dozing). A cut-off score ≥ 10 was used to define EDS. Pittsburgh Sleep Quality Index (PSQI) was used to evaluate night-time sleep quality of patients in the past month [29]. The scale was “recommended” for screening and measuring severity of overall sleep problems in PD patients [27]. The PSQI scale shows strong correlation with SCOPA-Sleep [28]. Although the PSQI does include five items to be filled out by the caregiver, but those are not included in the total score. Nineteen individual items generate seven component scores (subjective sleep quality, sleep latency, sleep duration, habitual sleep efficiency, sleep disturbances, use of sleeping medication, and daytime dysfunction). The total score ranges from 0 to 21, and the higher the score, the worse the sleep quality. We distinguished between good and poor sleepers using a cut-off point > 5 with good sensitivity and specificity.

### Statistics

The participants were divided into male and female, and EOPD and LOPD patients. Continuous variables including age, AAO, level of education, duration of disease, Daily LED, H-Y stage, UPDRS I, UPDRS II, UPDRS III, HRSD and MMSE score were expressed as mean ± standard deviation or median (interquartile range). Use the Student’s T test for comparisons when the variables met the normal distribution, and Mann-Whitney U test for data that did not have a normal distribution. Categorical variables including motor subtype, motor fluctuation, dyskinesia, RBD, smoking, family history of PD, history of hypertension and diabetes mellitus, and use of drugs for PD were expressed as frequency (%) and were compared using the χ2 test or Fisher’s exact test. Multivariate logistic regression analysis was conducted to explore the risk factors of declined sleep quality and EDS in patients with PD according to sex and AAO stratifications. We included age, sex, AAO, disease duration, UPDRS I, UPDRS II, and UPDRS III score, motor subtype, daily LED, RBD, HRSD and MMSE in adjusted models. All *p* values were two-tailed, and a significance level of 0.05 was used. Statistical analysis was conducted using SAS version 9.2 (Cary, NC, USA).

## Results

### Demographic and clinical characteristics

Among the 586 patients with PD, there were 347 males (59.2%), 239 females (40.8%), 86 EOPD (14.7%) and 500 LOPD (85.3%) patients. The demographic and clinical characteristics of patients with PD are listed in Table 1. Most of our subjects had H&Y staging ≤2.5. In our study, 461 patients had H&Y 2.5 or lower, 103 patients had H&Y 3, and only 17 and 5 patients had 4 and 5 points, respectively. Thus, our results may be more reflective of the characteristics of patients in the initial and medium stages. There was no significant sex difference in PD patients in terms of AAO, H&Y stage, UPDRS I and II score, motor subtype, movement fluctuation, dyskinesia, family history of PD, concurrent disease, RBD, MMSE, depression score, dopamine drugs, and daily LED. The educational level [9.0 years (6.0–12.0) vs. 9.0 years (2.0–9.0), *p* <  0.001], the number of smokers [120 (34.6%) vs. 5 (2.1%), *p* <  0.001], the UPDRS III scores [24.0 (16.0–33.0) vs 22.0 (12.0–30.0), *p* = 0.004] were higher in men. There was no significant difference between EOPD and LOPD patients in terms of disease duration, H&Y stage, UPDRS III score, movement fluctuation, dyskinesia, smoking, RBD, depression score, dopamine drugs, and daily LED. The AAO of LOPD patients was 63.9 ± 7.4 years. LOPD patients had higher UPDRS I scores (*p* <  0.001) and UPDRS II scores (*p* = 0.001). The number of LOPD patients with hypertension was 122/500 (24.4%), which was significantly higher than that in EOPD patients (12/86, 14.0%) (*p* = 0.033). This cohort seems to have more severe cognitive impairment (*p* <  0.001). The AAO of EOPD patients was 45.1 ± 5.2 years, and more patients had a family history of PD than LOPD patients had (11/86, 12.8% vs 31/500, 6.2%, *p* = 0.029). The classification of motor subtypes was different between the two groups (*p* = 0.023). Patients with EOPD tend to present with tremor-based motor symptoms (22, 25.6% vs 88, 17.6%), while patients with LOPD are more likely to present with rigidity (50, 58.1% vs 363, 72.6%) (*p* = 0.033).

**Table 1:** Baseline characteristics of 586 patients with Parkinson’s disease

Characteristic^a^	Male (*n* = 347)	Female (*n* = 239)	*P*-value	EOPD (*n* = 86)	LOPD (*n* = 500)	*P*-value
Age, years	65.7 ± 10.2	64.1 ± 9.03	0.049	49.5 ± 6.8	67.7 ± 7.5	< 0.001
Age at onset of PD, years	61.5 ± 10.2	60. 6 ± 9.0	0.294	45.1 ± 5.2	63.9 ± 7.4	< 0.001
Education, years	9.0 (6.0–12.0)	9.0 (2.0–9.0)	< 0.001	9.0 (6.0–12.0)	9.0 (6.0–12.0)	0.030
Duration of disease, months	36.0 (22.0–72.0)	36.0 (16.0–60.0)	0.047	41.0 (17.8–84.0)	36.0 (18.0–63.8)	0.433
Daily LED, mg/d	300 (0.0–400.0)	200 (0.0–400.0)	0.480	225 (0.0–450.0)	250 (0.0–400.0)	0.773
Hoehn-Yahr stage	2.0 (1.5–2.5)	2.0 (1.5–2.5)	0.109	2.0 (1.5–2.5)	2.0 (1.5–2.5)	0.249
UPDRS I	3.0 (2.0–5.0)	3.0 (2.0–4.0)	0.790	3.0 (1.0–4.0)	3.0 (2.0–5.0)	< 0.001
UPDRS II	9.0 (8.0–14.0)	10.0 (7.0–14.0)	0.240	9.0 (5.0–12.0)	11.0 (7.0–14.0)	0.001
UPDRS III	24.0 (16.0–33.0)	22.0 (12.0–30.0)	0.004	21.0 (14.5–32.0)	24.0 (15.0–32.8)	0.430
Motor subtype			0.104			0.023
TD	62 (17.8)	52 (21.7)		22 (25.6)	88 (17.6)	
A-R	252 (72.8)	161 (67.3)		50 (58.1)	363 (72.6)	
MX	33 (9.4)	26 (11)		14 (16.3)	49 (9.8)	
Motor fluctuation	47 (13.5)	39 (16.3)	0.330	11 (12.8)	75 (15.0)	0.583
Dyskinesia	14 (4.0)	15 (6.3)	0.219	4 (4.7)	25 (5.0)	0.890
Cigarette smoking	120 (34.6)	5 (2.1)	< 0.001	23 (26.7)	102 (20.4)	0.185
Family history of PD	25 (7.2)	17 (7.1)	0.966	11 (12.8)	31 (6.2)	0.029
History of hypertension	82 (23.6)	52 (21.8)	0.596	12 (14.0)	122 (24.4)	0.033
History of diabetes mellitus	24 (6.9)	14 (5.9)	0.609	3 (3.5)	35 (7.0)	0.222
Use of levodopa	209 (60.2)	146 (61.1)	0.835	45 (52.3)	310 (62.0)	0.090
Use of dopamine agonists	98 (28.2)	57 (23.8)	0.347	22 (25.6)	133 (26.6)	0.843
Use of MAOBI	30 (8.6)	15 (6.3)	0.290	10 (11.6)	35 (7.0)	0.137
Use of amantadine	51 (14.7)	31 (13.0)	0.554	15 (17.4)	67 (13.4)	0.318
Use of COMTI	12 (3.5)	3 (1.3)	0.097	0 (0.0)	15 (3.0)	0.104
RBD	126 (36.2)	72 (30.0)	0.110	24 (27.9)	173 (34.2)	0.253
HRSD	7.0 (3.0–13.0)	7.0 (3.0–15.0)	0.446	7.0 (2.0–13.0)	7.0 (3.0–14.0)	0.682
MMSE	28.0 (26.0–29.0)	27.0 (26.0–29.0)	0.055	29.0 (27.0–30.0)	28.0 (26.0–29.0)	< 0.001

^a^Continuous variables are expressed as mean ± standard deviation or as median (interquartile range). Categorical variables are expressed as frequency (percent)

*Abbreviations:* *PD* Parkinson’s disease, *LED* Levodopa-equivalent dose, *UPDRS I* Score of first part of Unified Parkinson Disease Rating Scale, *UPDRS II* Score of second part of Unified Parkinson Disease Rating Scale, *UPDRS III* Score of third part of Unified Parkinson Disease Rating Scale, *TD* Tremor Dominant, *A-R* Akinetic-rigid, *MX* Mixed, *MAOBI* Monoamine oxidase-B inhibitor, *COMTI* Catechol-O-methyltransferase inhibitor, *RBD* Rapid eye movement sleep behavior disorder, *HRSD* Hamilton Rating Scale for Depression, *MMSE* Mini-mental State Examination

### Sleep evaluation

The daytime sleepiness and night-time sleep quality of PD patients with different sexes and AAO are shown in Table 2. The ESS scores of male PD patients were significantly higher than those of female patients (*p* <  0.001). PD patients with ESS score ≥ 10 accounted for 24.1%, of which male patients (102/347, 29.4%) were more common than female patients (39/239, 16.3%) (*p* <  0.001). There was no difference in ESS score between EOPD and LOPD patients. The PSQI scores were higher in LOPD patients (*p* = 0.006). PSQI > 5 was found in 53.7% of PD patients, among whom, LOPD (276/500, 55.2%) was significantly more common than EOPD (35/86, 40.7%) (*p* <  0.013). There was no difference in night-time sleep quality between male and female PD patients in the past month.

**Table 2:** Sleep scales results for the sex stratification and age at onset stratification in PD patients

Variables^a^	Male *n* = 347	Female *n* = 239	*P*-value	EOG *n* = 86	LOG *n* = 500	*P*-value
ESS	6.0 (3.0–10.0)	4.0 (1.0–8.0)	< 0.001	4.5 (2.0–9.0)	5.5 (2.0–9.0)	0.203
ESS ≥ 10, n%	102 (29.4)	39 (16.3)	< 0.001	20 (23.3)	121 (24.2)	0.850
PSQI	6.0 (4.0–9.0)	6.0 (4.0–9.0)	0.761	5.0 (2.0–8.0)	6.0 (4.0–9.0)	0.006
PSQI> 5, n%	182 (52.4)	129 (54.0)	0.716	35 (40.7)	276 (55.2)	0.013

^a^Continuous variables are expressed as mean ± standard deviation or as median (interquartile range). Categorical variables are expressed as frequency (percent)

*Abbreviations:* *EOG* Early-onset group, *LOG* Late-onset group, *ESS* Epworth Sleepiness Scale, *PSQI* Pittsburgh Sleep Quality Index

### Risk factors of ESS and PSQI in PD patients based on sex and AAO stratifications

Multivariate logistic regression analysis showed that in all PD patients, the risk factors of ESS were male sex, disease duration, and HRSD score. According to sex stratification, HRSD score and AAO were risk factors for ESS in male and female PD patients, respectively. According to AAO stratification, HRSD score was the only risk factor for ESS in EOPD patients, while in LOPD patients, male sex, disease duration, and HRSD score were risk factors (Table 3). In all patients with different sexes and AAO, HRSD score was the common risk factor related to PSQI (Table 4).

**Table 3:** Significant predictors of ESS as binary variables according to sex stratification and age at onset stratification from multivariate logistic regression analysis in PD patients

Variables	Odds ratio (95% confidence interval)	*P*-value
**Overall model**		
Male sex	2.163 (1.398–3.346)	0.001
Disease duration	1.010 (1.001–1.018)	0.023
HRSD	1.040 (1.016–1.065)	0.001
**Male model**		
HRSD	1.047 (1.047–1.017)	0.002
**Female model**		
Age at onset	1.196 (1.003–1.425)	0.046
**EOG model**		
HRSD	1.060 (1.000–1.125)	0.051
**LOG model**		
Male sex	2.362 (1.465–3.808)	< 0.001
Disease duration	1.011 (1.002–1.020)	0.017
HRSD	1.038 (1.011–1.067)	0.006

*Abbreviations:* *ESS* Epworth Sleepiness Scale, *PD* Parkinson’s disease, *HRSD* Hamilton Rating Scale for Depression, *EOG* Early onset group, *LOG* Late-onset group

**Table 4:** Significant predictors of PSQI as binary variables according to sex stratification and age at onset stratification from multivariate logistic regression analysis in PD patients

Variables	Odds ratio (95% confidence interval)	*P*-value
**Overall model**		
HRSD	1.098 (1.069–1.128)	< 0.001
**Male model**		
HRSD	1.104 (1.063–1.146)	< 0.001
**Female model**		
HRSD	1.103 (1.056–1.153)	< 0.001
**EOG model**		
HRSD	1.084 (1.021–1.151)	0.008
**LOG model**		
HRSD	1.111 (1.007–1.146)	< 0.001

*Abbreviations:* *PSQI* Pittsburgh Sleep Quality Index, *PD* Parkinson’s disease, *HRSD* Hamilton Rating Scale for Depression, *EOG* Early onset group, *LOG* Late-onset group

## Discussion

Our study focused on analyzing the clinical characteristics of daytime sleepiness and night-time sleep quality in PD patients of different sexes and AAO.

PD patients with different sexes and AAO show certain specific clinical phenotypes [10, 14, 30]. Similar findings were found in our study. The prevalence of PD in male patients is 1.45 times higher than that of female patients. Male patients had more severe motor symptoms. This benign phenotype of female may be related to differences in sex hormones. Some studies have revealed that estrogen has a neuroprotective effect on the dopaminergic system, thus reducing the risk of PD and the rate of disease progression in women [31]. Sex differences in genes are also involved in different clinical characteristics. The low expression of some GABA receptor genes observed in women may be related to the prevalence of tremor as the first symptom and the increased expression of proinflammatory genes detected in women may explain that they are more likely to be affected by depression and anxiety [32], although no differences in HRSD scores were found between men and women in our study. We found that women had worse cognitive function than men, although there was no statistical significance. Women have lower levels of education than men, which may explain the difference. A recent study on the relationship between occupational stress and PD risk shows that high job requirements seem to increase the risk of PD in men, especially among highly educated men [33], which may partly explain men’s susceptibility to PD.

Age is the most important risk factor for PD. We found that patients with LOPD had higher UPDRS I and II scores, had relatively poor cognitive function and were more likely to present with the AR subtype. This is consistent with previous researches, suggesting that patients with LOPD show more axial symptoms, including abnormal posture and gait, as well as NMSs [13, 34]. In contrast, the disease progresses slowly in EOPD, usually with family history and earlier motor complications [35]. About 19% of EOPD patients in our study had a family history. A study based on autopsy of PD patients showed that EOPD patients have a higher proportion of lifelong use of amantadine and dopamine agonists and a higher incidence of dyskinesia [36]. However, there was no difference in dopaminergic drugs and motor complications between the two groups in our study, which may be related to the different samples and drug selection habits of doctors in different countries and regions.

Sleep disturbance is one of the most important NMSs that affect the quality of life in PD patients. Sleep deterioration is an important marker of the prodromal phase of PD. Some studies have shown that PD patients may have difficulty falling asleep, broken sleep, early awakening, lack of sleep, EDS, snoring, nightmares, and hallucinations. EDS in PD patients is associated with unexpected, sudden sleep or sleep seizures, which can increase driving risk and reduce quality of life [37]. EDS is also a marker of dopamine loss, and a lower [123I] FP-CIT (DaTscan) value in the caudate nucleus is associated with a higher EDS score [38]. In our study, 24.1% of PD patients reported EDS, and men had more and severer daytime sleepiness than women had. Multivariate logistic regression analysis showed that male sex, disease duration, and depression were associated with EDS in all PD patients and LOPD patients in particular; depression was associated with EDS in men and EOPD patients; and AAO was associated with EDS in female patients. Previous studies have shown that predictive factors for EDS include male sex, disease duration and severity of PD, dose of dopamine agonist, apnea or hypopnea index, and BMI [39, 40], which are similar to those in our study. NMSs such as depression, fatigue, pain, cognitive impairment, cardiovascular disease, urinary tract disorders, and thermoregulatory disorders may also lead to EDS [41]. A longitudinal study also showed that PSG parameters in EDS are more common in individuals with depression [42]. In PD patients, serotonergic, noradrenergic and cholinergic neurons in the brainstem also act as arousal systems to maintain awake, and dysfunction of these neurons in PD patients with depression can lead to EDS [43]. In terms of AAO, Guo et al. reported that the prevalence of EDS or fatigue in LOPD patients was higher than in EOPD patients [16]. However, we found no difference in the frequency and severity of EDS between LOPD and EOPD patients, which is the same as a previous longitudinal study [41]. We found that the AAO is a risk factor for EDS in female patients. In EOPD patients, EDS was associated with depression only but neither sex nor disease duration.

Besides experiencing EDS, PD patients may have other nocturnal sleep disorders. In our study, 53.7% of PD patients had poor night-time sleep quality. It is generally believed that older women have lower subjective sleep quality than older men have, although PSG results showed a better sleep structure [44]. No difference was found between men and women in our study. Based on AAO stratification, the overall sleep quality of patients with LOPD was worse than that of EOPD patients, which is similar to the results of Mahale et al. [45]. Sleep disorders may increase with aging. Sleep efficiency began to decrease significantly after the age of 60 years, and the age-related sex difference was more significant in elderly patients [46]. We found that LOPD patients had higher UPDRS I and II scores, indicating that LOPD patients had more mental, behavioral and emotional problems such as depression, hallucinations, and decreased daily activity, and more NMSs that may be closely related to sleep quality. Multivariate logistic regression analysis showed that depression was the common risk factor for poor night-time sleep quality in all PD patients in our study, regardless of sex or early or late onset of PD. Around 30–40% of PD patients may experience depression [47]. In PD patients with depression, REM latency decreases, total REM sleep time and REM density increase, and slow-wave sleep decreased [48], which was closely related to nocturnal sleep, especially insomnia. It is suggested that the sleep disorders in PD patients are not only the results of dopamine deficiency and neurodegeneration of ascending awakening system, but also the results of damage to the serotoninergic and noradrenergic systems related to pain and depression [49]. However, the HRSD scale contains three sleep items. It may affect the scores of the PSQI and ESS, the effect of depression on sleep needs to be elucidated in more objective ways.

Our study had several limitations. Firstly, a healthy control group was not included. Secondly, most of our subjects had H&Y 2.5 or less, reflecting the selection bias of our study. Our results may be more reflective of the characteristics of patients in the initial and medium stages. Thirdly, this study was conducted using rating scales, without PSG or MSLT. As a self-rated scale, the ESS scale does not include information from a caregiver or partner. Thus, the risk may be underestimated. The PSQI does not adequately cover other sleep disturbances such as RLS, RBD or PLMs. While the PSQI scale shows strong correlation with SCOPA-Sleep, but not with PSG. So further studies that combine the PD-specific questionnaire and PSG to evaluate the sleep disorders more comprehensively are needed.

## Conclusion

In summary, our study shows that EDS and poor night-time sleep quality are more common in PD patients. Male patients are more likely to have EDS than female patients have, and LOPD patients have worse night-time sleep quality. Male sex, disease duration, and depression are the main risk factors for EDS in PD patients, while depression is a predictive factor of poor night-time sleep quality in all PD patients, whether they are male or female, and have early- or late-onset PD. Clinicians should pay attention to sleep disorders of PD patients, especially in male and LOPD patients. Depression should be routinely evaluated in PD patients with sleep disorders.

## Data Availability

All data generated or analyzed during this study are included in this published article. Some or all data or models generated or used during the study are available from the corresponding author by request.

## Abbreviations

AAO: Age at onset
EDS: Excessive daytime sleepiness
EOPD: Early-onset Parkinson’s disease
ESS: Epworth Sleepiness Scale
HRSD: Hamilton Rating Scale
LED: Levodopa equivalent dose
MMSE: Mini-mental State Examination
NMS: Nonmotor symptom
PD: Parkinson’s disease
PSQI: Pittsburgh Sleep Quality Index
RBD: Rapid eye movement sleep behavior disorder
REM: Rapid eye movement
RLS: Restless leg syndrome
UPDRS: Unified Parkinson’s Disease Rating Scale
